# Whole Brain Amide Proton Transfer Weighted Imaging in Children With Obstructive Sleep Apnea

**DOI:** 10.1002/brb3.70808

**Published:** 2025-09-02

**Authors:** Guisen Lin, Weiting Tan, Shaojun Zhang, Qin Yang, Yijiang Zhuang, Shijie Hu, Dongxia Mo, Kan Deng, Wenhong Ye, Hongwu Zeng

**Affiliations:** ^1^ Department of Radiology Shenzhen Children's Hospital Shenzhen China; ^2^ Department of Medical Imaging Shenzhen Luohu People's Hospital Shenzhen China; ^3^ Department of Respiratory medicine Shenzhen Children's Hospital Shenzhen China; ^4^ Philips Healthcare Guangzhou China

## Abstract

**Purpose:**

To investigate alteration of brain amide proton transfer weighted (APTw) signals in children with obstructive sleep apnea (OSA) and to assess the association of APTw signals in different brain regions with cognitive impairment.

**Methods:**

This prospective study was conducted from September 2021 to December 2023. Forty‐six children with mild to severe OSA and 20 non‐OSA volunteers with matched age and gender underwent a whole brain APTw imaging scan. The APTw signals of 29 regions of the brain were compared between the OSA group and volunteers with Fisher's least significant differences post‐hoc analysis or the Kruskal–Wallis test with the Steel‐Dwass test. The correlation of the APTw signals of different brain regions and cognitive assessment scores was analyzed with Pearson's correlation analysis or Spearman's correlation analysis.

**Results:**

The APTw signals in the white matter of the inferior frontal gyrus, the white matter of the angular gyrus, and the thalamus of children with mild OSA and volunteers were significantly lower than that of children with moderate‐severe OSA. The APTw signals of the gray matter of the supramarginal gyrus, the gray matter of the lingual gyrus, the corona radiata, and the genu of corpus callosum in children with moderate‐severe OSA were significantly lower than that of volunteers. The APTw signals of the frontal‐parietal‐temporal regions, hippocampus, and corona radiata were significantly associated with cognitive assessment scores.

**Conclusions:**

APTw imaging could be used for detecting frontal‐parietal‐temporal regions and hippocampus damage in children with OSA, which possibly explain cognitive impairments associated with OSA.

## Graphical abstract

The amide‐proton‐transfer weighted (APTw) signals of the frontal‐parietal‐occipital region, hippocampus, and corona radiata in children with obstructive sleep apnea (OSA) were significantly decreased and were positively associated with cognitive assessment scores. It suggested that the APTw signal could be an imaging biomarker for assessment of neurocognitive impairment in children with OSA.

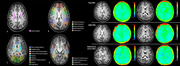

**Abbreviations**
APTw: amide‐proton‐transfer weighted
FIQ: full‐scale IQ
MG: mild obstructive sleep apnea group
MSG: moderate to severe obstructive sleep apnea group
OAHI: obstructive apnea‐hypopnea index
OSA: obstructive sleep apnea
PRI: Perceptual Reasoning Index
PSI: Processing Speed Index
VCI: Verbal Comprehension Index
WMI: Working Memory Index

## 1 Introduction

Obstructive sleep apnea (OSA) is a common respiratory disorder affecting 1.2% to 5.7% of the pediatric population (Marcus et al. 2012). A growing body of evidence indicates that long‐term untreated OSA is associated with neurocognitive dysfunction (Wu et al. 2020) and growth problems (Salorio et al. 2002). Irreversible cognitive impairments may happen in children with OSA (Gozal 2008), which can significantly impact their long‐term development since childhood is a critical period for nervous system maturation. Additionally, untreated childhood OSA considerably increases the utilization of health resources (Reuveni et al. 2002). These factors highlight the need for early recognition, assessment, and treatment of children with OSA.

Hypoxia and neuronal injury commonly occur in vulnerable areas of the brain in patients with OSA (Cooper 1999). Animal models have demonstrated that chronic intermittent hypoxia can increase reactive oxygen species production and oxidative stress, resulting in cortical neuronal cell apoptosis (Xu et al. 2004). MR imaging could be used to assess brain structural and functional changes in children with OSA to explore the relationship between brain changes and cognitive decline. Studies have found reduced gray matter volume in children with OSA in the frontal, temporal, and parietal lobes (Philby et al. 2017); MR spectroscopy reveals altered neuronal metabolities in the frontal cortex and hippocampus in severe OSA children who exhibit impaired verbal skill IQ and executive functions (Halbower et al. 2006).

Amide proton transfer weighted (APTw) imaging is a promising molecular MR imaging technique that detects endogenous mobile proteins (Zhou et al. 2019). It has been widely applied in neuroimaging for assessment of ischemic stroke (Lin et al. 2018), neurodegenerative disease (Wang et al. 2019), and brain tumors (Zhou et al. 2003). A recent animal study demonstrated that APTw imaging could reveal proteostasis disturbance in the hippocampus related to neural injury induced by sleep deprivation (Zhao et al. 2022). We hypothesized that the APTw signal of OSA children would decrease in vulnerable areas of the brain due to neuronal death, potentially correlating with cognitive impairment. The purpose of our study was to investigate the APTw signal alteration of OSA children in different brain regions and to evaluate the association of APTw signal in these regions with cognitive impairment.

## 2 Methods

### 2.1 Participants

This study was approved by the Ethics Committees of Shenzhen Children's Hospital (#2022036 and #2023080902). Guardians of the participants signed informed consent forms to participate in this project. From September 2021 to December 2022, children (age 6–18) suspected of OSA were recruited for the patient group. The inclusion criteria for the patient group were OSA diagnosed with obstructive apnea‐hypopnea index (OAHI) ≥ 1 times/h and no previous treatment for OSA. Age‐ and gender‐matched non‐OSA volunteers without snoring were recruited from outpatient clinics. They attend clinics for medical consultations other than OSA, such as developmental concerns, workups of endocrinopathy, and vomiting. A Pediatric Sleep Questionnaire of 22 items (Savini et al. 2019) was conducted for children in healthy volunteers to exclude OSA. The exclusion criteria for participants were: history of cerebral infarction/hemorrhage, central nervous system infection, CO poisoning, epilepsy, traumatic brain injury, or mental disorders; claustrophobia or metallic implants that were contraindicated to MR scan; Poor MR imaging quality. Children in the patient group were divided into a mild OSA group (MG; 1 ≤ OAHI < 5 times/h) and a moderate‐to‐severe OSA group (MSG; OAHI ≥ 5 times/h).

### 2.2 Polysomnography Recording and Pediatric Sleep Questionnaire

Children with OSA underwent full‐night PSG recording (Somnoscreen Plus Tele PSG, Somnomedics GMBH, Germany) for over 7 h, including an electroencephalogram, an electrooculogram, a chin electromyogram, and an electrocardiogram in the sleep laboratory. Thoracic‐abdominal strain gauges, transcutaneous finger pulse oximeters, and oronasal cannulas were monitored to record episodes of apneas and hypopneas. All the sleep data was analyzed by a medical electrophysiology technologist according to the American Academy of Sleep Medicine sleep scoring manual (Berry et al. 2012). Guardians and the volunteers filled out the Pediatric Sleep Questionnaire of 22 items with the assistance of a well‐trained staff. Volunteers should have a score less than 0.33 (Chervin et al. 2000) to be included.

### 2.3 Cognitive Function Assessment

We used the Wechsler Intelligence Scale for Children, fourth edition, Chinese version index scores (Yang et al. 2013) for cognitive function assessment. Participants were evaluated by an experienced psychologist, who was blinded to the status of the participants, in a quiet room without the company of guardians. The assessment results were summed into a four‐index score and a full‐scale IQ (FIQ). The four‐index score includes the Verbal Comprehension Index (VCI), Perceptual Reasoning Index (PRI), Working Memory Index (WMI), and Processing Speed Index (PSI).

### 2.4 MR Protocol and Image Analysis

An MR scan was performed on a 3 Tesla MR system (Ingenia, Philips Healthcare, Best, the Netherlands) without anesthesia. Whole brain axial APTw imaging was acquired using the 3D TSE‐DIXON approach with a continuous saturation duration of 2 s and RF saturation amplitude of 2 µT. Saturation frequency offsets were implemented at 3.5 ppm, 3.5 ± 0.8 ppm, ‐3.5 ppm, ‐3.5 ± 0.8 ppm, and ‐1560 ppm. At 3.5 ppm, three Z‐spectral images are acquired using a different echo time shift on the order of 0.5 milliseconds. The other imaging parameters were as follows: repetition time (TR), 6120 ms; echo time (TE), 7.8 ms; field of view (FOV), 230 mm × 180 mm × 168 mm; spatial resolution, 256 × 256; slice thickness, 6 mm; sensitivity encoding (SENSE) factor, 1.6; total scan time for whole brain APT imaging, 13–16 min. A co‐registered axial 3D magnetization‐prepared rapid gradient echo technique (MPRAGE) was also performed for identification of anatomical structure.

**FIGURE 1:**
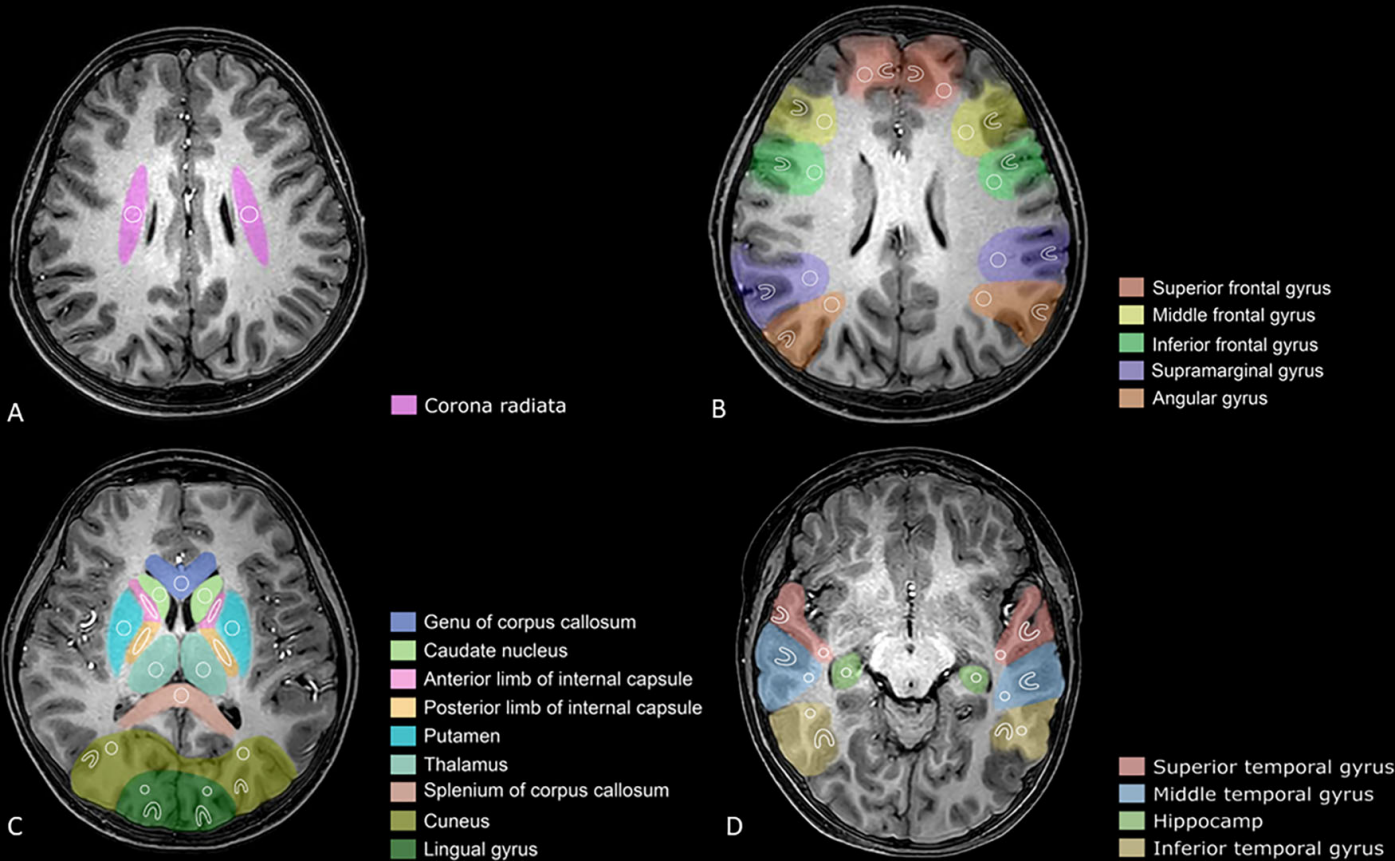
Examples of the definition of the regions of interest on T1‐MPRAGE images.

The APTw imaging was analyzed with the Philips Intellispace Portal software in the MR workstation, which was constructed by the MRT asymmetry at the offsets of ± 3.5 ppm. Twenty‐nine regions of interest (ROIs) were drawn by 2 radiologists with over 5 years of experience in neuroimaging who were blinded to the status of the participants (Figure 1). These 29 ROIs were the superior frontal gyrus, middle frontal gyrus, inferior frontal gyrus, superior temporal gyrus, middle temporal gyrus, inferior temporal gyrus, supramarginal gyrus, angular gyrus, cuneus of occipital lobe, lingual gyrus, corona radiata, genu of corpus callosum, splenium of corpus callosum, anterior limb of internal capsule, posterior limb of internal capsule, caudate, putamen, thalamus, and hippocampus. For the ROIs of the frontal, temporal, parietal, and occipital lobes, both white matter and gray matter were drawn separately.

### 2.5 Statistical Analysis

Data was analyzed using the SPSS (Version 22.0, IBM Corporation, Chicago, IL). All the ROIs were drawn bilaterally, and the mean was reported. For quantitative data, if they are normally distributed, the mean with standard deviation was reported; otherwise, the median with interquartile range was reported. The chi‐square test was used for comparison of the gender difference between non‐OSA volunteers, MG, and MSG. For comparison of other clinical data, neurocognitive assessment scores, and APTw signals between volunteers, MG, and MSG, one‐way analysis of variance with Fisher's least significant differences post‐hoc analysis was used if normally distributed; otherwise, the Kruskal–Wallis test with Steel‐Dwass test was used. For comparison of polysomnography data between MG and MSG, student's *t* test was used if normally distributed; otherwise, the Mann–Whitney *U* test was used. For the analysis of the correlation between APTw signals with the neurocognitive assessment, Pearson's correlation analysis was used if normally distributed; otherwise, Spearman's correlation analysis was used.

Intraclass correlation coefficients for the 29 ROIs drawn by 2 radiologists were calculated.

## 3 Results

### 3.1 Clinical Information, Polysomnography Results, and Neurocognitive Assessment

**FIGURE 2:**
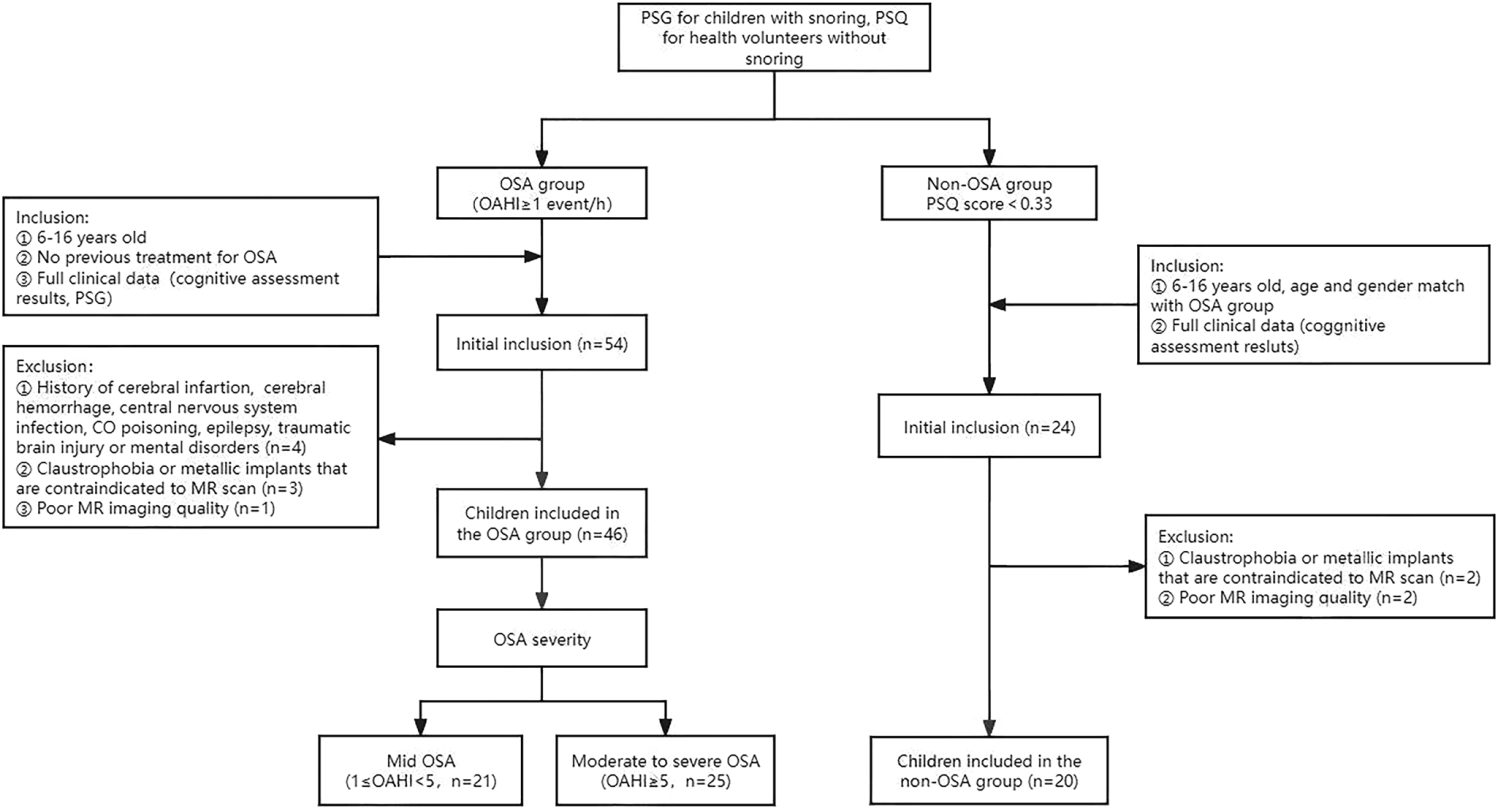
The recruitment process of children with OSA and non‐OSA volunteers.

Figure 2 shows the recruitment process of the patient group and non‐OSA volunteers. Initially, 54 consecutive children were recruited into the patient group. After reviewing the medical history, two children with epilepsy, one child with a previous intracranial infection, and one child with a previous traumatic brain injury were excluded. Three children with claustrophobia and one child with poor MR imaging quality were excluded, leaving 46 children in the patient group for analysis. There were 21 children with mild OSA (median age, 8.41 years; 13 males) and 25 children with moderate to severe OSA (median age, 9.20 years; 19 males). For non‐OSA volunteers, 24 children were recruited initially. After excluding 2 children with claustrophobia and 2 children with poor MR imaging quality, 20 children (median age, 9.29 years; 15 males) remained for analysis. Table 1 summarizes the basic clinical characteristics, polysomnography, and neurocognitive assessment of the volunteers and patient group. There was no significant difference in age or gender among the three groups. Patients in the MSG had significantly higher BMI than that of the volunteers and MG (*p* < 0.001). Patients in the MSG had worse apnea‐hypopnea index (AHI), obstructive apnea index (OAI), OAHI, sleep efficiency (SE), arousal index, and minimal SpO^2^ than patients in the MG (all *p* < 0.01). The FIQ, VCI, and PSI were significantly higher in volunteers than that of MG and MSG (all *p* < 0.01). However, there was no significant difference in FIQ, VCI, and PSI between patients in MG and MSG. For PRI and WMI, there was no significant difference among the three groups.

**TABLE 1:** Demographic, sleep, and neurocognitive characteristics of non‐OSA volunteers and children with OSA.

Variables	Non‐OSA volunteers (*n* = 20)	MG (*n* = 21)	MSG (*n* = 25)	*p* value
**Gender (F/M)**	15/5	13/8	19/6	0.52^a^
**Age (years)**	9.29 (8.57, 10.69)	8.41 (7.65, 9.99)	9.20 (7.99, 10.91)	0.15^b^
**BMI (kg/m^2)**	15.00 (14.63, 16.23)^!^	15.73 (14.40, 20.80)^!^	22.00 (18.55, 24.90)^*^	< 0.001^b^
**SE (%)**	—	78.28 ± 2.56	68.84 ± 2.33	0.009^c^
**Minimal SpO2 (%)**	—	91.00 (89.50, 93.00)	85.00 (80.50, 87.00)	< 0.001^d^
**OAHI (events/hr)**	—	1.53 (1.18, 2.75)	6.57 (5.82, 11.38)	< 0.001^d^
**AHI (events/hr)**	—	1.70 (1.20, 2.75)	6.9 (5.5, 11.7)	< 0.001^d^
**OAI (events/hr)**	—	0.30 (0.15, 0.70)	1.9 (1.1, 3.5)	< 0.001^d^
**FIQ**	109.50 (102.25, 113.00)^*!^	100.00 (90.50, 103.00)	91.00 (86.50, 102.00)	< 0.001^e^
**VCI**	104.75 ± 1.52^*!^	92.33 ± 2.39	88.76 ± 1.93	< 0.001^b^
**PRI**	111.85 ± 2.05	110.95 ± 2.78	104.80 ± 2.59	0.095^b^
**WMI**	99.10 ± 2.18	95.57 ± 2.94	94.16 ± 2.70	0.41^b^
**PSI**	108.50 ± 1.83^*!^	97.19 ± 3.44	96.68 ± 2.39	0.004^b^

*Note*: Data is presented as mean ± SD or median with interquartile range. ^*^ vs. MG; ^!^ vs. MSG. ^a^ Chi‐Square test; ^b^ Kruskal–Wallis test with Steel‐Dwass test; ^c^ student's *t* test; ^d^ Mann–Whitney *U* test; ^e^ one‐way analysis of variance (ANOVA) with Fisher's least significant differences post‐hoc analysis.

**Abbreviations**: AHI, apnea hypopnea index; BMI, body mass index; FIQ, full‐scale IQ; MG, mild OSA group; MSG, moderate‐severe OSA group; OAHI, obstructive apnea hypopnea index; OAI, obstructive apnea index; PRI, perceptual reasoning index; PSI, processing speed index; SE, sleep efficiency; VCI, verbal comprehension index; WMI, working memory index.

### 3.2 APTw Signals in Different Regions of the Brain

**FIGURE 3:**
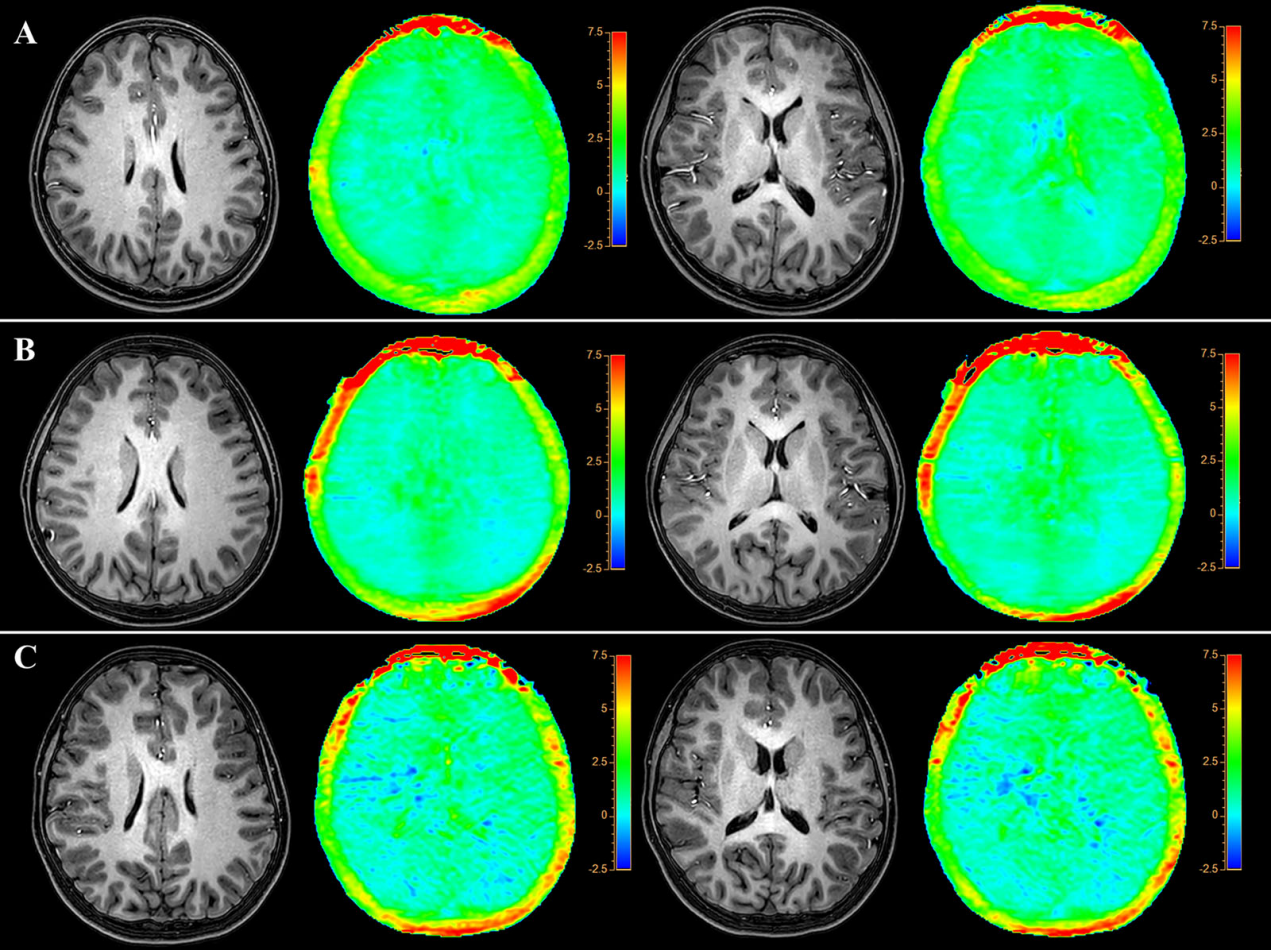
Representative 3D‐MPRAGE and APTw images from three children of **(A)** Non‐OSA volunteers, **(B)** MG **(B)**, and **(C)** MSG.

Figure 3 shows the representative 3D‐MPRAGE and APTw images from 3 children in the non‐OSA volunteers, MG, and MSG, respectively. Table 2 summarizes the APTw signals of the 29 regions of the brain among three groups. Patients in the MSG had significantly lower APTw signals in the gray matter of the inferior frontal gyrus, caudate, and hippocampus than that of MG and non‐OSA volunteers. The APTw signals in the white matter of the inferior frontal gyrus, white matter of the angular gyrus, and the thalamus of MG and MSG were significantly lower than that of volunteers. The APTw signals of the gray matter of the supramarginal gyrus, the gray matter of the lingual gyrus, the corona radiata, and the genu of the corpus callosum in MSG were significantly lower than those of non‐OSA volunteers. There was no significant difference in APTw signals of other regions of the brain among the three groups. The intraclass correlation coefficients for non‐OSA volunteers, MG, and MSG were 0.77 (0.37, 0.93), 0.82 (0.48, 0.94), and 0.80 (0.45, 0.94) respectively, which showed good consistency between 2 radiologists.

**TABLE 2:** Comparation analysis of APT values between non‐OSA volunteers and children with OSA.

Brain regions			Non‐OSA volunteers (*n*=20)	MG (*n*=21)	MSG (*n*=25)	*p* value
**Frontal lobe**	**Superior frontal gyrus**	Gray matter	1.12 ± 1.02	1.27 ± 0.08	1.18 ± 0.07	0.47^a^
		White matter	0.89 (0.71, 1.06)	0.85 (0.60, 1.02)	0.87 (0.67, 1.17)	0.88^b^
	**Middle frontal gyrus**	Gray matter	1.25 ± 0.09	1.25 ± 0.09	1.19 ± 0.08	0.78^a^
		White matter	0.87 (0.64, 1.02)	0.68 (0.53, 0.91)	0.74 (0.47, 0.90)	0.34^b^
	**Inferior frontal gyrus**	Gray matter	1.19 ± 0.05^！^	1.19 ± 0.05^！^	0.91 ± 0.03^*^	0.003^b^
		White matter	0.96 (0.90, 1.03)^*！^	0.79 (0.66,0.99)	0.77 (0.64,0.94)	0.001^b^
**Temporal lobe**	**Superior temporal gyrus**	Gray matter	1.39 ± 0.85	1.27 ± 0.09	1.44 ± 0.09	0.86^a^
		White matter	1.25 ± 0.08	1.28 ± 0.08	1.27 ± 0.09	0.98^a^
	**Middle temporal gyrus**	Gray matter	1.33 ± 0.09	1.29 ± 0.07	1.31 ± 0.07	0.95^a^
		White matter	1.31 ± 0.09	1.30 ± 0.09	1.28 ± 0.08	0.98^a^
	**Inferior temporal gyrus**	Gray matter	1.58 ± 0.08	1.41 ± 0.11	1.48 ± 0.06	0.39^a^
		White matter	1.26 (1.03,1.67)	1.38 (0.80,1.56)	1.40 (1.10,1.53)	0.73^b^
**Parietal lobe**	**Supramarginal gyrus**	Gray matter	1.08 ± 0.08^！^	0.93 ± 0.07	0.75 ± 0.06	0.005^a^
		White matter	0.82 ± 0.06	0.72 ± 0.06	0.68 ± 0.05	0.22^a^
	**Angular gyrus**	Gray matter	0.84 ± 0.06	0.76 ± 0.06	0.67 ± 0.05	0.11^a^
		White matter	0.71 ± 0.05^*！^	0.51 ± 0.06	0.50 ± 0.05	0.013^a^
**Occipital lobe**	**Cuneus**	Gray matter	0.67 (0.60, 0.80)	0.72 (0.46,0.84)	0.64 (0.49, 0.83)	0.73^b^
		White matter	0.48 (0.35, 0.61)	0.57 (0.38,0.69)	0.45 (0.28, 0.56)	0.29^b^
	**Lingual gyrus**	Gray matter	0.77 ± 0.05^！^	0.67 ± 0.08	0.58 ± 0.05	0.049^b^
		White matter	0.66 ± 0.05	0.62 ± 0.07	0.54 ± 0.05	0.32^a^
**Basal ganglia area**	**Corona radiata**		0.93 ± 0.09^!^	0.79 ± 0.05	0.631 ± 0.05	0.015^b^
	**Genu of corpus callosum**		1.19 ± 0.14^！^	1.39 ± 0.13	1.62 ± 0.11	0.04^a^
	**Splenium of corpus callosum**		1.46 ± 0.17	1.62 ± 0.12	1.32 ± 0.17	0.42^b^
	**Anterior limb of internal capsule**		1.62 ± 0.09	1.55 ± 0.09	1.60 ± 0.10	0.878^a^
	**Posterior limb of internal capsule**		1.36 (1.09, 1.64)	1.28 (0.75, 1.39)	1.18 (0.83, 1.52)	0.34^b^
	**Caudate nucleus**		1.48 ± 0.11^！^	1.58 ± 0.10^!^	1.85 ± 0.07^*^	0.013^a^
	**Putamen**		1.28 ± 0.08	1.23 ± 0.09	1.37 ± 0.09	0.52^a^
	**Thalamus**		1.53 (1.09, 1.82)^*！^	1.25 (0.97, 1.33)	1.23 (1.06, 1.38)	0.044^b^
	**Hippocampus**		1.91 ± 0.11^!^	1.75 ± 0.06^!^	1.38 ± 0.07^*^	<0.001^a^

*Note*: Data is presented as mean ± SD or median with interquartile range. ^*^ vs. MG; ^!^ vs. SG. ^a^ one‐way analysis of variance (ANOVA) with Fisher's least significant differences post‐hoc analysis; ^b^ Kruskal–Wallis test with Steel‐Dwass test.

**Abbreviations**: MG, mild OSA group; MSG, moderate‐severe OSA group.

### 3.3 Correlation of APTw Signals With the Neurocognitive Assessment Result

Table 3 summarizes the correlation of the APTw signals of different brain regions with the neurocognitive assessment result for children in non‐OSA volunteers, MG, and MSG. The APTw signal of the gray matter of the inferior frontal gyrus was positively correlated with VCI (*r* = 0.44, *p* < 0.001), PRI (*r* = 0.41, *p* = 0.001), and FIQ (*r* = 0.48, *p* < 0.001). The APTw signal of the white matter of the inferior frontal gyrus was positively correlated with VCI (*r* = 0.33, *p* = 0.007), PRI (*r* = 0.26, *p* = 0.034), PSI (*r* = 0.34, *p* = 0.005), and FIQ (*r* = 0.39, *p* = 0.001). The APTw signal of the gray matter of the supramarginal gyrus was positively correlated with VCI (*r* = 0.43, *p* < 0.001), PRI (*r* = 0.31, *p* = 0.011), WMI (*r* = 0.25, *p* = 0.44), PSI (*r* = 0.30, *p* = 0.015), and FIQ (*r* = 0.46, *p* < 0.001). The APTw signal of the white matter of the angular gyrus was positively correlated with VCI (*r* = 0.32, *p* = 0.009), PRI (*r* = 0.27, *p* = 0.029), WMI (*r* = 0.32, *p* = 0.01), and FIQ (*r* = 0.43, *p* < 0.001). The APTw signal of the gray matter of the lingual gyrus was positively correlated with PRI (*r* = 0.24, *p* = 0.049) and FIQ (*r* = 0.28, *p* = 0.024). APTw signal of the corona radiata was positively correlated with VCI (*r* = 0.30, *p* = 0.016) and FIQ (*r* = 0.34, *p* = 0.006). The APTw signal of the hippocampus was positively correlated with VCI (*r* = 0.38, *p* = 0.002). No significant correlation between the APTw signals of other regions and neurocognitive assessment results were found.

**TABLE 3:** Correlation analysis between APT signal intensity of different brain regions and neurocognitive assessment scores.

Brain regions	VCI	PRI	WMI	PSI	FIQ
Superior frontal gyrus gray matter	***r = 0.436***	***r = 0.414***	*r = 0.195*	*r = 0.221*	***rs = 0.482***
	***p < 0.001***	***p = 0.001***	*p = 0.117*	*p = 0.075*	***p < 0.001***
Inferior frontal gyrus white matter	***rs = 0.331***	***rs=0.262***	*rs=0.203*	***rs=0.341***	***rs=0.387***
	***p = 0.007***	p = 0.034	*p = 0.102*	***p = 0.005***	***p = 0.001***
Supramarginal gyrus gray matter	***r = 0.429***	***r = 0.311***	***r = 0.248***	***r = 0.297***	***rs = 0.457***
	***p < 0.001***	***p = 0.011***	***p = 0.044***	***p = 0.015***	***p < 0.001***
Angular gyrus white matter	***r = 0.317***	***r = 0.269***	***r = 0.317***	*r = 0.208*	***rs = ‐0.425***
	***p = 0.009***	***p = 0.029***	***p = 0.010***	*p = 0.093*	***p < 0.001***
Lingual gyrus gray matter	*r = 0.170*	***r = 0.243***	*r = ‐0.164*	*r = 0.186*	***rs = 0.278***
	*p = 0.171*	***p = 0.049***	*p = 0.188*	*p = 0.135*	***p = 0.024***
Corona radiata	***r = 0.296***	*r = 0.232*	*r = 0.087*	*r = 0.209*	***rs = ‐0.338***
	***p = 0.016***	*p = 0.061*	*p = 0.485*	*p = 0.093*	***p = 0.006***
Genu of corpus callosum	*r = ‐0.124*	*r = ‐0.021*	*r = ‐0.186*	*r = ‐0.077*	*rs = ‐0.087*
	*p = 0.320*	*p = 0.867*	*p = 0.135*	*p = 0.540*	*p = 0.487*
Caudate nucleus	*r = ‐0.183*	*r = 0.030*	*r = ‐0.185*	*r = ‐0.064*	*r = ‐0.053*
	*p = 0.141*	*p = 0.809*	*p = 0.137*	*p = 0.607*	*p = 0.674*
Thalamus	*rs = 0.170*	*rs = 0.165*	*rs = 0.031*	*rs = 0.203*	*rs = 0.216*
	*p = 0.173*	*p = 0.186*	*p = 0.805*	*p = 0.102*	*p = 0.082*
Hippocamp	***r = 0.378***	*r = 0.087*	*p = 0.034*	*r = 0.017*	*rs = 0.189*
	***r = 0.002***	*p = 0.487*	*p = 0.789*	*p = 0.891*	*p = 0.129*

*Note*: rs: Spearman's correlation analysis; *r*: Pearson's correlation analysis; *p* < 0.05 considered statistically and presented in bold.

**Abbreviations**: FIQ, full‐scale IQ; PRI, perceptual reasoning index; PSI, processing speeding index; VCI, verbal comprehension index; WMI, working memory index.

## 4 Discussion

In this study, we compared the APTw signals across different brain regions between non‐OSA volunteers and children with OSA. We further investigated the correlation between APTw signals and neurocognitive impairment in children with OSA. To our knowledge, this is the inaugural study to explore the whole brain APTw imaging in pediatric OSA patients. The results showed that children with OSA had decreased APTw signals in several regions of the brain, which correlated with neurocognitive impairment. This underscored the feasibility and capability of using APTw imaging for assessing cognitive impairments in children with OSA.

The APTw signal is predominantly affected by protein content and pH levels (Zhou et al. 2019), decreasing as protein content diminishes or pH levels fall. A previous study has shown that patients with OSA had decreased N‐acetylaspartate (NAA)/choline ratios in the white matter of the brain, suggesting neuronal loss or axonal injury (Kamba et al. 2001). A recent study showed that the APTw signal decreased in the hippocampus of rats subjected to sleep deprivation, which was negatively correlative with the expression of glucose‐regulated protein 78 and positively correlative with the surviving neurons (Zhao et al. 2022). This suggested that the decreased APTw signal was associated with decreased protein content due to neuronal death. Regarding pH, the accumulation of lactate has been detected in the deep white matter of patients with OSA during sleep (Kamba et al. 2001). However, no lactate signal was detected with MR spectroscopy in awake OSA patients (Kamba et al. 2003). As all children underwent MR scans while awake without sedation, we assumed the level of lactate was not increased in the brain during scanning. Therefore, the decrease of the APTw signal in children with OSA was most likely due to decreased protein content related to neural loss.

Patients with OSA have cellular damage in gray matter, white matter, and regions that are sensitive to oxygen supply (Baril et al. 2021). A large‐scale cohort study with 775 adults with OSA revealed that a lower mean arterial oxygen saturation was linked to reduced volumes in the frontoparietal cortex, hippocampus, thalamus, and caudate (Marchi et al. 2020). These volume reductions were associated with chronic cellular responses, such as neuronal death, glial loss, and reduced synaptic density. In pediatric patients with OSA, similar findings with reduced gray and white matter volumes in the frontal lobe (Hongbin et al. 2023, Yu et al. 2023), parietal lobe (Musso et al. 2020), and temporal lobe (Philby et al. 2017) were also observed. These regions are sensitive to oxygen supply and are thus vulnerable to hypoxic attack in OSA patients (Lim and Veasey 2010). Recent studies in children with OSA also showed damage in the white matter of the frontal lobe and thalamus resulting in abnormal topological structural connectivity, which might be the neurofunctional basis for impaired cognitive function (Tan et al. 2025). Our study's results showed that the APTw signal decreased in gray matter (inferior frontal gyrus, supramarginal gyrus, and lingual gyrus), white matter (inferior frontal gyrus and angular gyrus), hippocampus, corona radiata, genu of corpus callosum, and thalamus, indicating possible neural damage of these areas. It is noteworthy that a previous MR spectroscopy study of OSA patients showed a decreased NAA/choline ratio only in the white matter but not the gray matter of the brain (Kamba et al. 2001). However, due to the low spatiotemporal resolution of MR spectroscopy (Faghihi et al. 2017), incomplete separation of the cerebral gray matter from white matter may result in errors in measuring the NAA/choline ratio for the gray matter (Kamba et al. 2001). In contrast, APTw imaging could detect both changes in gray matter and white matter in patients with OSA, possibly contributing to its high spatiotemporal resolution.

Children with OSA tend to experience more severe cognitive impairment (Lo Bue et al. 2020). Compared with non‐OSA volunteers, children in the patient group had decreased FIQ, VIC, and PSI, aligning with Zhao's study of children (age ≥ 6 years) with OSA (Zhao et al. 2018). Chemical and structural cellular injury of the prefrontal cortex in patients with OSA has been implicated in certain patterns of cognitive impairment, especially for executive dysfunction (Beebe and Gozal 2002). The development of the frontal‐parietal‐temporal network that includes both the cortex and the white matter is related to visuo‐spatial working memory during childhood, and its impairment could lead to a decline of specific cognitive functions (Pearson 2019, Klingberg 2006). In our study, the APTw signal intensity in the frontal‐parietal‐temporal regions was significantly related to the cognitive assessment scores, suggesting that the APTw signal may be a possible imaging biomarker for assessment of neurocognitive impairment in children with OSA. In addition, the APTw signal intensity of the hippocampus and corona radiata were significantly associated with a few cognitive assessment scores. The hippocampus and parahippocampal cortex are interconnected with multiple brain areas in the frontal‐parietal lobes, which play a crucial role in cognitive processes (Opitz 2014). The hippocampus is also well‐known for its role in declarative memory. However, we did not identify a correlation between the APTw signal intensity of the hippocampus with WMI or FIQ. A coronal section of the hippocampus would be better for measurement of the APTw signal, which was not performed in our study. This might be a reason for not detecting the association. The corona radiata is vulnerable to microvascular damage, and its microstructural integrity has been shown to correlate with cognitive performance (Badji et al. 2019). Our finding suggested that the damage of corona radiata may be related to certain cognitive impairments of children with OSA.

One important limitation of the present study was the lack of polysomnography for the non‐OSA volunteers. However, the Pediatric Sleep Questionnaire of 22 items we used for excluding OSA is a reliable test that has a correct classification for over 85% of subjects (Chervin et al. 2000). The second limitation was that we did not include the scanning of the cerebellum due to the limited time for scanning. Previous studies have shown gray matter volume loss of the cerebellum in patients with OSA (Zimmerman and Aloia 2006). Further studies are needed to examine the changes of APTw signal in the cerebellum and its correlation to cognitive impairment in patients with OSA. However, it should be noticed that the repeatability of the APTw signal is excellent in the supratentorial locations but poor in the infratentorial locations (Lee et al. 2020). Third, the small sample size may restrict the generalizability of the findings. Fourth, we did not account for the duration of untreated OSA that may influence the extent of neural damage. Fifth, the reliance on FIQ and its subscales (VCI, PRI, WMI, PSI) may overlook domain‐specific deficits (e.g., executive function, attention). The inclusion of additional tests (e.g., BRIEF or Trail Making Test) could provide a more comprehensive view of cognitive impairment. Last, an additional coronal section of the hippocampus may be better to quantify ATPw signal intensity, which was not performed in our study. Additionally, the 6‐mm slice thickness of APTw imaging may limit detection of subtle changes in small structures like the hippocampus. However, reducing slice thickness would dramatically increase the scanning time.

In conclusion, the results of this study showed that children with OSA had decreased APTw signal intensity in multiple brain regions, possibly related to protein decreases induced by neural death. The APTw signals of the frontal‐parietal‐occipital region, hippocampus, and corona radiata were positively associated with cognitive assessment scores, suggesting that the APTw signal could be an imaging biomarker for assessment of neurocognitive impairment in children with OSA.

## Author Contributions

**Guisen Lin**: conceptualization, data curation, formal analysis, writing – original draft, and funding acquisition. **Weiting Tan**: conceptualization, data curation, formal analysis, and writing – original draft. **Shaojun Zhang**: conceptualization, formal analysis, data curation, and writing – original draft. **Qin Yang**: conceptualization and data curation. **Yijiang Zhuang**: conceptualization and data curation. **Shijie Hu**: conceptualization and data curation. **Dongxia Mo**: conceptualization and data curation. **Kan Deng**: conceptualization and software. **Wenhong Ye**: conceptualization, writing – review and editing and supervision. **Hongwu Zeng**: conceptualization, writing – review and editing, funding acquisition, and supervision.

## Conflicts of Interest

The authors declare no conflict of interests.

## Peer Review

The peer review history for this article is available at https://publons.com/publon/10.1002/brb3.70808.

## Data Availability

The data that support the findings of this study are available on request from the corresponding author. The data are not publicly available due to privacy or ethical restrictions.
